# Transient Hyperammonemia Seen in Post Seizure Activity: A Series of Six Case Reports

**DOI:** 10.51894/001c.5780

**Published:** 2017-02-02

**Authors:** Justin T. Daughtry, Kevin M. Boehm

**Affiliations:** 1 Onslow Memorial Hospital. Department of Emergency Medicine, Jacksonville, NC; 2 St. Mary Mercy Hospital, Department of Emergency Medicine Faculty and Graduate Medical Education. Livonia, MI; Michigan State University, College of Osteopathic Medicine. East Lansing, MI

**Keywords:** diagnostics, clinical markers, hyperammonemia, seizure

## Abstract

Elevations in serum ammonia levels (i.e., hyperammonemia) have often been interpreted as signs of liver failure or errors in metabolism. The purpose of this series of case studies is to evaluate a trend in both the markedly elevated levels of ammonia, along with its rapid clearance in patients with seizures. These patient cases each occurred in a community-based, academic emergency department in the metropolitan Detroit area that provided care to approximately 70,000 patients during a four-year period. These six patient cases had each been found with observed seizure activity in which an initial and repeat ammonia in the emergency department was ordered. In all six cases, there was an initial elevation in ammonia with rapid subsequent clearance of ammonia. As demonstrated in this series of case studies, transient hyperammonemia levels may occur within the clinical context of seizure activity. With further research, it may be confirmed to be a differential diagnostic marker to delineate new onset or recurrent seizures in the ED.

## INTRODUCTION

A new onset seizure is often difficult to diagnosis by an emergency medicine (EM) clinician unless the seizure actually occurs in front of them until an electroencephalogram (EEG) has been taken. The information that the emergency department (ED) physician has to rely on comes from family or the patient themselves, physical exam (e.g., new intraoral lesions), and based on diagnostic tests to rule out other non-epileptic form of seizure like activity.^1^ Inpatient or outpatient EEGs can be arranged to confirm diagnosis of seizure, but clinicians have asked whether there a lab value that may aid in these diagnoses.

This series of case reports demonstrates the possible link between transient hyperammonemia and seizure activity. Although hyperammonermia is usually associated with hepatic encephalopathy, inborn errors of metabolism, and disruption in the urea cycle, the authors hope to demonstrate from this series of case reports that ammonia levels as a laboratory marker for seizure activity can potentially provide physicians with another tool to better diagnosis new onset seizures in the ED.

### Case Reports

### Case 1

This patient was a 46-year-old male who per EMS had a witnessed grand-mal seizure prior to arrival to the ED. The patient’s history was obtained primarily from the EMS providers, who stated they suspected a possible overdose due to a medication bottle found near the patient near time of seizure. The patient’s vital signs were as follows: heart rate (HR) 118, blood pressure (BP) 136/64, respiratory rate (RR) 20, and oxygen saturation of 100% was measured on a non-rebreather oxygen mask. His initial Glasgow Coma Scale (GCS) score (on range from 3 (maximal neurological deficit) to 15 (no deficits) was 6 at arrival to the ED. During primary survey, however, his neurological status later improved to a GCS score of 9. The patient was found to be oriented to person only, and responded to pain.

Physical exam showed pupils 4mm and reactive bilaterally, a minor laceration on the outer lower lip, lungs were clear, and heart rate was tachycardic (i.e., elevated). The patient had pulses in all extremities with an external fixator on the right lower extremity from a recent orthopedic surgery at another hospital. The patient had a past medical history that included: seizure disorder, bipolar disorder, depression, suicidal attempt, schizophrenia, anxiety, hypertension, diabetes peripheral neuropathy, hyperlipidemia, osteoarthritis, chronic low back pain. His social history included past alcohol abuse and cocaine abuse.

The patient’s oral home medications included: aspirin 81 mg. daily, citalopram 20 mg. daily, pregabalin 75 mg. twice a day, felodipine 10 mg. daily, quetiapine 300 mg. twice a day, iron 325 mg. daily, clonidine 0.1 mg. twice a day, valproate 500 mg. in the morning and 1500 mg. at night, and folate 1 mg. daily.

After his initial assessment in the ED, the patient was given lorazapam 2mg. intravenously (IV) at 0744 hours. His labs studies demonstrated elevated lactic acid and creatinine levels with a low potassium and an anion gap of 33. His ammonia was elevated to 145 umol/l with normal liver function testing (normal ammonia is under 50 umol/l). Other medications which were administered included potassium chloride 40 mEq. orally, and valproate 1500 mg. orally at 1302 hours. The patient continued to have a prolonged postictal state (i.e., altered state of consciousness after an epileptic seizure) and he was admitted to the hospital.

During his hospital stay, a computerized tomography (CT) of the brain was performed showing no acute pathology. An ultrasound of the abdomen was performed which showed gallstones without evidence for acute cholecystitis but poor views of the liver and pancreas obtained due to the patient’s physique. A hepatobillary (HIDA) scan showed a normal hepatobiliary system with normal response of gallbladder to cholecystokinin (CCK) stimulation following administration of CCK with the ejection faction to be 77%. An EEG was performed showing the presence of slow frequency in the theta range varying from 4-7 cycles per second. No epileptiform activity was seen. The conclusion of the consulting neurologist was that the EEG was mildly abnormal because of the presence of slow theta frequency with this abnormality suggestive of mild cerebral dysfunction. The patient stayed in the hospital for two days and was discharged with a diagnosis of seizure. This case showed the ammonia clearing to 23 umol/L in approximately 13 hours (see Table 1).

**Table 1:** Highlighted lab work for the six cases presented that was performed and the approximate times the repeat labs were drawn*

	**Case 1**	**Case 2**	**Case 3**	**Case 4**	**Case 5**	**Case 6**
						
**Initial Values**						
**SODIUM** [135-145] mmol/L	141	143	139	137	140	143
**POTASSIUM** [3.5-5.0] mmol/L	**3.2**	4	**3.3**	**3.4**	3.1	3.8
**CHLORIDE** [95-108] mmol/L	96	97	96	96	101	100
**CARBON DIOXIDE** [24-32] mmol/L	**12**	**10**	**19**	**15**	**16**	25
**ANION GAP** [8-16]	**33**	**36**	**24**	**26**	**23**	**18**
**BUN** [10-25] mg/dL	24	N/A	7	12	16	14
**CREATININE** [0.9-1.3] mg/dL	**2.2**	N/A	1	1.2	1.13	1.11
**AST** [15-35] IU/L	14	**132**	**73**	n/a	31	N/a
**ALT** [30-65] IU/L	25	**85**	36	n/a	45	N/a
**AMMONIA** [18-50] umol/L	**145**	**290**	**111**	**277**	**96**	**86**
						
** Repeat Values**	**Approx. 13 hrs. later**	**Approx. 3 hrs. later**	**Approx. 16 hrs. later**	**Approx. 3 hrs. later**	**Approx. 3 hrs. later**	**Approx. 2 hrs. later**
**SODIUM** [135-145] mmol/L	142	139	145	141	138	139
**POTASSIUM** [3.5-5.0] mmol/L	3.9	4.5	3.5	**3.4**	3.7	3.7
**CHLORIDE** [95-108] mmol/L	105	106	109	108	107	104
**CARBON DIOXIDE** [24-32] mmol/L	27	26	22	27	25	31
**ANION GAP** [8-16]	10	7	14	6	6	4
**AMMONIA** [18-50] umol/L	23	19	29	22	47	47

*Please note, abnormally high or low values are listed in **bold font**. (Normal lab value ranges are listed in brackets).

### Case 2

This patient presented as a 47-year-old male with a past medical history of cardiac disease, including a myocardial infarction and untreated hypertension. His surgical included history of a hernia repair, and he had a social history of cocaine and alcohol abuse. The patient had been brought to the ED by several members of his family. Per family member reports, the patient usually drank between 12 and 24 beers each day. The patient had reportedly stopped drinking 24 hours prior to his arrival to the ED. Per the family, the patient had also been “shaking” all day and had seemed to have become increasingly confused.

During the ED triage process, the patient had a witnessed grand-mal seizure that lasted for approximately 60 seconds. During that time, an IV line was established and the patient was given 2mg. of lorazapam for the seizure. As the seizure subsided, he appeared postictal but eventually began experiencing hallucinations. His was slightly hypertensive with a BP of 148/103, a HR 136, RR of 18, temperature of 37 C and pulse oximetry of 95% on room air. On physical exam, the patient was diaphoretic, with no pain to palpation on the chest or abdomen. His lung sounds were clear bilaterally and he was able to move all four extremities to pain.

However, he was unable to follow commands, attempted to make incoherent speech, and seemed neither alert nor oriented. Patient was administered a two-liter IV fluid bolus of normal saline. The patient’s labs were then drawn and the results are listed in Table 1. These labs showed the patient to have an elevated anion gap, likely secondary to a lactic acidosis, elevated ammonia, elevated liver enzymes, and thrombocytopenia. A CT of the head was also performed with no acute findings.

The patient’s abnormal LFT’s and thrombocytopenia were thought to be secondary to his chronic alcoholism, and he was started on the hospital ethanol withdrawal pathway. This protocol included treatment with IV lorazapam, multivitamins, folic acid, and thiamine. The authors decided to treat the patient’s elevated ammonia with oral lactulose since his mental status had improved at the time they received the lab results. The lactic acidosis was treated with an initial IV fluid bolus of two liters with plans to repeat the lactate to see if it was clearing.

Approximately three hours after the first lab set was drawn, the patient’s electrolytes, ammonia, and lactic acid levels were rechecked along with a total bilirubin (Table 1). The ammonia level had returned to normal before the patient’s system having had enough time for the oral lactulose treatment to start working since it must first change the flora of the gut and cause the resulting diarrhea.^2^ In light of these circumstances, it was concluded that the seizure, and not the suspected liver disease, must be the cause of the patient’s transient hyperammonemia.

The patient continued to improve in the ED with normalization of vital signs and he was admitted to a hospital unit for further monitoring. During his hospital course, an infectious hepatitis panel blood test was performed that was negative for hepatitis A, B, and C. An ultrasound of the liver was performed which showed the liver to be mildly echogenic. During his hospital stay, the patient had no subsequent elevations of his ammonia level and no further seizure activity was noted.

### Case 3

This patient was a 49-year-old male seen in the ED for witnessed seizure activity. Emergency medical service personnel had been called to the patient’s house for an impending seizure and actually witnessed the patient seizing in the hallway. The seizure had stopped prior to his arrival in the ED, where the patient had another witnessed grand-mal seizure once in the ED. The patient began foaming at the mouth, not protecting his airway, with a GCS score of 3. The decision was made to intubate the patient. The patient was given 4mg of midazolam and 80mg of rocuronium. After the intubation, the patient was placed on a propofol drip for sedation and administered IV hydromorphone 1mg. twice approximately one hour apart.

The patient’s past medical history included seizure disorder, alcohol abuse, pancreatitis, and a prior history of seizures during ethanol withdrawal. He had no surgical history and took no recorded home medications. His social history was positive for alcohol abuse. The physical exam showed a normal head-eyes-ears-nose throat, lungs and abdomen. The patient neurological exam prior to intubation showed an unresponsive patient with a GCS score of 3 and foaming at the mouth.

He was admitted to the intensive care unit (ICU) for respiratory failure and seizures. This patient was never started on any medications for the elevated ammonia, having no prior history of hepatic encephalopathy. With only IV fluids and monitoring, the patient’s ammonia was cleared to normal levels in a little over 24 hours.

The patient had an extended stay in the ICU and on the general hospital floor. Neurology service was consulted, although no EEG was performed since the etiology of the seizures was thought to be due to alcohol withdrawal. The patient was initially started on phenytoin for the seizures, but per neurology's recommendations the patient was switched to levetiracetam due to the elevation in his ammonia and liver function tests (LFT). During the patient’s hospital stay, he developed pneumonia and a GI bleed that were all successfully treated. Once stable, the patient was transferred to a patient rehabilitation unit with a psychology consult placed for the patient’s suspected psychosis. The patient was finally discharged home 2.5 months after his initial ED visit with a normal ammonia level.

### Case 4

This patient was a 28-year-old male with a known seizure disorder. He was brought to the ED for evaluation after his brother had witnessed his seizure which he described as a grand–mal seizure at home. The patient only apparently experienced one seizure and was now presenting postictal. Per the family the patient had noncompliance with the medication carbamazepine, which he had been prescribed for twice a day use. The patient’s past medical history is positive only for seizures, with no surgical history. He had a past history of tobacco use with a total 5 pack year history, and smoked marijuana on a daily basis with the last use the day prior to arrival.

The patient’s exam showed a somewhat confused patient despite a GCS of 15. HEENT, cardiac, lung, and abdominal exam were negative for acute findings. Neurological exam showed no focal deficits. His labs showed an elevated ammonia level at 227 and elevated lactic acid. Patient also had a serum anion gap, indicating some form of electrolyte disturbance. During the patients ED stay, the patient only received a dose of carbamazepine 500mg orally and IVF. As shown in Table 1, the patient’s ammonia and lactic cleared in 3 hours. The patient was observed in the ED and with return to normal mental status before being discharged home.

### Case 5

Patient was a 37-year-old man, who was non English speaking, with a past medical history for seizures and brought to the ED for a witnessed seizure by family. Most of the history was given by EMS. Upon arrival to the ED, this patient continued to have seizures. The patient was given 2mg lorazapam followed by 2 mg of midazolam. The seizures stopped and he gradually became more alert. Over the course of the ED visit, the patient's neurologic status continued to improve. The patient was examined with the help of a phone interpreter, although he remained unclear regarding the seizure medication he took. Valprioic acid, phenytoin, and carbazempem lab levels were obtained, with each failing to show such medications detected.

The results from a CT of the head were negative for acute pathology. The patient's labs showed an initial lactic acid at 15.8, an ammonia level of 96 with an anion gap of 23. Labs were repeated in approximately three hours showing resolution of lab abnormalities. During that time, the patient was given 4 liters of Normal saline and 500mg of levetiracetam intravenously. The family eventually arrived in the ED to be at the patient’s bedside. The patient was completely alert and oriented to his baseline per family and was discharged home.

### Case 6

The patient in this case was a 24-year-old male. According to his parents, the patient was a healthy individual with no past medical history or social history. He had been traveling with his family in the back seat of the car when he became unresponsive and “started shaking” per his parents. The emergency medicine personnel who were on scene indicated that the patient had experienced a witnessed generalized seizure and was unresponsive until arrival to the ED.

In the ED, the patient appeared to be postictal but slowly became more arousible, capable of answering questions for himself. As the patient's mental status improved, he became combative, requiring an initial dose of 2mg of lorazapam then a second dose of 6mg of lorazapam. The labs for this patient showed an elevated ammonia level at 86, lactic acid of 8.1 and an anion gap of 18. Urinary drug screen, alcohol, and CT of the head were also negative.

After discussion with the family, the patient was then admitted to the hospital due to the possibility of a first seizure. An MRI of the brain and EEG were both performed and returned with normal results. Patient was seen by neurology and was discharged home on phenytoin with a discharge diagnosis of generalized seizures.

Table 1 highlights the lab work that was performed for each of these patients and the specific times that the initial and subsequent labs were drawn. Please note that abnormal values are listed in bold font. As this was a retrospective case series, there were no criteria for timing of initial or repeat lab draws followed. Labs were drawn upon evaluation of the patients and repeated as indicated by clinicians’ judgments.

As shown in Table 1, each of the patients had initially elevated ammonia levels. Even though repeat labs were drawn at various times, each showed decreased patient ammonia levels. Of note, Cases 2, 4, 5 showed ammonia levels of 290, 277, 96 respectfully, with each of these patients’ repeat labs performed around 3 hours later and showing normal ammonia lab values (i.e., 19, 22, 47, respectively). Also of interest, Case 6, with an initial ammonia of 86, showed a repeat normal ammonia level approximately two hours later.

## DISCUSSION

The differential for hyperammonemia can include many etiologies such as congenital deficiencies of the urea cycle enzymes, hepatic encephalopathy’s, Reye syndrome, as well as several other metabolic disorders. Each of these etiologies can result in sustained ammonia levels and require treatment for resolution. Transient hyperammonemia etiologies, and their effects, have been discussed far less often in the medical literature.

Transient hyperammonemia of the newborn (THAN) is primarily seen in premature infants, the exact cause unknown but potentially lethal. The treatment involves protein avoidance, adequate caloric support, arginine supplementation, and pharmacological priming of alternative pathways for nitrogen excretion. Once the serum ammonia level is normalized, there is no further risk of new episodes of hyperammonemia and normal intake of protein can occur.^3^

Transient Hyperammonemia has also been studied in patients on chronic valproate treatment for seizure prevention. One of the potential side effects of valproate treatment is elevated ammonia levels. The cause of this is thought to be related to a deficiency of the amino acid derivative carnitine. By depleting carnitine from the body, there is a subsequent decrease in the amount of free acetyl CoA, which is required to activate carbamyl phosphate synthetase I, the first enzyme in the urea cycle. However, elevated ammonia levels have still been shown in patients with high protein diets supplementation of oral carnitine to decrease serum ammonia in patients on valproate.^4^ A similar therapeutic effect of oral carnitine on ammonia levels was supported during a study of patients with hepatic encephalopathy.^5^ In patients with hepatic encephalopathy, neuronal activity improved after a single short course of IV L-carnitiine and acetyl-L-carnitine (ALCAR).^5^

During these cases, transient hyperammonemia was discovered in the context of witnessed seizure activity. In these cases, it was found that over half of these witnessed seizure patients had measured hyperammonemia in the absence of hepatic disease. The patients’ hyperammonemia levels in these cases had a short clearance time of the ammonia of one to three hours, which is unexpected if the elevation had been caused by valproate use or hepatic encephalopathy.^6^

The possible mechanisms of action causing hyperammonemia in some instances could also be from muscle.^7^ In a previous study of cyclists undergoing strenuous exercise elevated levels of ammonia could be detected.^8^ Most seizures involve a considerable amount of muscle activation, and may explain rises in ammonia levels.^6^ Although these types of transient elevations may seem to be harmless, one study showed that a long-lasting alteration in protein kinase A occurred after a single episode of hyperammonemia. These ammonia alterations were shown to affect signal transductions in the brain and the blood, altering levels of cGMP and possibly affecting platelet aggregation, portal hypertension and hypotension. Additionally, such changes may be involved in other manifestations of liver disease.^6^

## CONCLUSION

True seizure activity that is not witnessed by a treating physician can be difficult to diagnosis in absent of EEG testing. Using transient hyperammonemia lab values could, however, be used as a practicable solution to identifying true seizure activity in ED settings. Since few studies involving this phenomenon have been conducted, further investigations of the causes of elevated ammonia levels and the effect of these elevations is also needed. As demonstrated in this series of case studies, transient hyperammonemia levels may occur within the clinical context of seizure activity. With further research, it may be confirmed to be a diagnostic marker to delineate new onset or recurrent seizures in the ED.

### Conflict of Interest

The authors declare no conflict of interest.
